# Virulence Is More than Adhesion and Invasion Ability, an In Vitro Cell Infection Assay of Bovine *Mycoplasma* spp.

**DOI:** 10.3390/microorganisms13030632

**Published:** 2025-03-11

**Authors:** Elhem Yacoub, Daniel Kos, Murray Jelinski

**Affiliations:** 1 Department of Large Animal Clinical Sciences, Western College of Veterinary Medicine, University of Saskatchewan, Saskatoon, SK S7N 5A2, Canada; elhem.yacoub@usask.ca; 2 Institute for Microbial Systems and Society, University of Regina, Regina, SK S4S 0A2, Canada; daniel.kos@usask.ca

**Keywords:** bovine, mycoplasma, MDBK, adhesion, invasion

## Abstract

*Mycoplasma bovis* is the most common mycoplasma associated with cattle diseases worldwide. However, other seemingly less virulent *Mycoplasma* spp. such as *M. bovigenitalium* and *M. bovirhinis* have also been associated with mycoplasmosis. The study objective was to compare the adhesion and cellular invasion characteristics of these bovine *Mycoplasma* spp. using Madin–Darby Bovine Kidney (MDBK) epithelial cells. MDBK cells were separately infected with 12 *M. bovis* strains and one strain each of *M. bovigenitalium* and *M. bovirhinis*. Following infection, a gentamicin protection assay was performed and the cells lysed at 6 and 54 h post-infection. The MDBK cell lysates were cultured for *Mycoplasma* spp. and qPCR was used to estimate the average number of *Mycoplasma* bacterial cells that infected each MDBK cell (Myc/Cell ratio). Confocal and electron microscopy studies using *M. bovis* mNeonGreen strain were also performed. All 14 *Mycoplasma* strains multiplied within the MDBK cells, a finding confirmed by microscopy studies of the *M. bovis* mNeonGreen strain. Unexpectedly, the *M. bovis* strains, obtained from diseased and asymptomatic cattle and bison, had lower Myc/Cell ratios than *M. bovirhinis* and *M. bovigenitalium* strains. These findings suggest that the ability for mycoplasmas to invade and replicate within host cells does not account for the differences in virulence between species.

## 1. Introduction

Mycoplasmas belong to the *Mollicutes* class, which are characterized by the lack of a cell wall. Lacking a cell wall results in a pleomorphic shape and confers intrinsic antimicrobial resistance to antibiotics such as the β-lactams that disrupt bacterial cell wall formation [1]. Mycoplasmas are ubiquitous and responsible for numerous pathologies of humans and livestock [1,2]. Bovine mycoplasmas have been isolated from many different tissues and organs and thus a spectrum of clinical signs are associated with bovine mycoplasmosis [3,4,5,6,7,8]. *M. bovis* is considered the most pathogenic of all bovine mycoplasmas, being associated with bronchopneumonia, mastitis, polyarthritis, otitis, and genital disorders [3,4,5,6,7,8], resulting in high economic losses and animal welfare concerns [9]. Although *M. bovis* is the primary mycoplasma associated with bovine respiratory disease (BRD), others such as *M. dispar* and *M. bovirhinis*, *M. bovigenitalium*, *M. arginini*, and *M. canis* have been recovered from the respiratory tracts of BRD calves [10]. *M. bovirhinis* is commonly found in the respiratory tracts of healthy cattle [11,12], and has been associated with clinical cases of BRD [13,14], whereas *M. bovigenitalium* has been linked to bovine endometritis [15]. Complicating matters, it is not uncommon to find a mixed mycoplasma population in samples obtained from healthy and sick cattle. Mixed populations of *M. bovis*, *M. bovirhinis*, and *M. canis* have been recovered from bronchoalveolar lavages of calves with and without BRD [16].

Biofilm formation as well as host cell adhesion and subsequent invasion contribute to the virulence, environmental persistence, dissemination, and survival of *M. bovis* [7,17]. Mycoplasma virulence factors and the mechanisms of pathogenicity have been reviewed elsewhere [18,19,20]. Briefly, pathogenesis begins with adhesion to mammalian cells, leading to cellular invasion. Arguably the most studied of the virulence factors is the family of variable surface lipoproteins (Vsps), which not only facilitate host cell adherence but also assist in evading the host’s immune system [7,20]. Additional well-studied adhesins include α-enolase, the VpmaX protein, NADH oxidase (Nox), and the TrmFO protein [20]. Of particular note is mycoplasmas’ ability to invade white and red blood cells, allowing them to evade the host’s immune system and facilitate dissemination within the animal. Although *M. bovis* does not produce conventional bacterial toxins, reactive oxygen and nitrogen species are involved in the pathogenesis of mycoplasma pneumonia. Other virulence factors are involved in suppressing the host’s production of interferon-γ and tumor necrosis factor-α.

Only three genome sequences of each *M. bovirhinis* and *M. bovigenitalium* have been publicly released [13,21,22], whereas over 600 *M. bovis* genomes are available in the NCBI database. These genomes along with genetically modified strains have contributed to a growing body of knowledge related to the biology of *M. bovis*. For example, the development of a universal fluorescence expression tool has facilitated the in vitro and in vivo study of *M. bovis*–host interactions [23]. Mechanisms mediating some of these interactions such as apoptosis, adherence, and cytotoxicity have also been studied [24,25,26,27,28]. Intracellular localization of *M. bovis* has been demonstrated in vitro with varying bovine cell types such as blood [29,30,31,32,33], nasal turbinate, lung, tracheal embryonic [29,34], as well as mammary gland epithelial cells [35]. The Madin–Darby Bovine Kidney (MDBK) cell line has also been shown to support a range of mycoplasmas, including *M. bovis* [36,37,38]. The gentamicin protection assay in conjunction with culture and confocal fluorescent microscopy has proven to be a useful technique for studying mammalian cell–mycoplasma interactions [31,32,33,34,35,36,39]. However, there are few contemporary studies that have investigated these interactions in non-*M. bovis* species such as *M. bovirhinis* and *M. bovigenitalium*. In 1974, Thomas et al. [40] demonstrated that *M. dispar* and *M. bovirhinis* infected fetal tracheal explant cultures, but only *M. dispar* had a cytotoxic effect. Regarding *M. bovigenitalium*, only one study has shown its capacity to cause cytopathic effects on calf, pig, and monkey kidney cell cultures [41].

The objective of our time course study was to describe the adhesion and invasion of, and multiplication within, MDBK cells by 14 bovine mycoplasma strains of *M. bovis*, *M. bovirhinis*, and *M. bovigenitalium*. The strains were chosen based on their different molecular and epidemiological backgrounds. We hypothesized that the more ‘virulent’ *Mycoplasma* spp. are, the more they would adhere to, invade, and replicate in the MDBK cells. A gentamicin protection assay was performed with culture and qPCR to assess the outcomes, with confocal and electron microscopy used to confirm the results.

## 2. Materials and Methods

Briefly, MDBK cells (also known as NBL-1), obtained from the American Type Culture Collection (ATCC CCL-22), were infected with 12 strains of *M. bovis* and one strain each of *M. bovigenitalium* and *M. bovirhinis*. All strains had undergone antimicrobial susceptibility testing to confirm sensitivity to gentamicin. The cells were then subjected to a gentamicin protection assay, which killed the extracellular mycoplasma population. Gentamicin was removed by washing and the cells incubated for either 6 or 54 h post-infection. Following MDBK cell lysis, lysate samples were collected and cultured for mycoplasmas and the amount of mycoplasma and host cell DNA quantified by qPCR. We also experimented with infection parameters, changing the multiplicity of infection (MOI) from 10 to 50, and increasing infection times from 3 to 24 h. Confocal and scanning electron microscopy were used to visualize invasion and replication within the MDBK cells.

### 2.1. Bacterial Strains, Media, and Culture

The 12 *M. bovis* strains included the type strain PG45 (ATCC 25523), three fluorescent strains mCherry (OC7), mKO2 (OK1), and mNeonGreen (ON8) (courtesy of Dr. Lucía Manso-Silván, CIRAD, Montpellier, France), and eight Canadian clinical isolates from the lungs, joints, and nasopharynges of cattle and bison of varying health status (asymptomatic and dead), see Table 1. The *M. bovirhinis* field strain (MP 212-A NTC 3) and *M. bovigenitalium* type strain (ATCC 19852) were also assessed. For simplicity, all *Mycoplasma* spp. (type strains, fluorescent strains, and field isolates) included in this study will herein be referred to as strains.

All bovine mycoplasma strains were originally stored in pleuropneumonia-like organism (PPLO) media with glycerol (20%, *v*/*v*) at −80 °C. The frozen suspensions of strains were thawed on ice then revived by culturing for 48 h at 37 °C with 5% CO_2_ in PPLO broth medium (Difco, Franklin Lakes, NJ, USA) supplemented with 10% yeast extract (Gibco, Waltham, MA, USA), 20% heat-inactivated horse serum (Gibco, USA), 0.5% sodium pyruvate (Sigma-Aldrich, Tokyo, Japan), 0.05% thallium acetate (Sigma-Aldrich, St. Louis, MO, USA), and 500 U/mL penicillin G (Sigma-Life Science, Burlington, MA, USA). Culture titers were calculated by performing 10-fold serial dilutions and plating on PPLO agar. After incubating for 72 h at 37 °C and 5% CO_2_, the fried egg-shaped *Mycoplasma* spp. colonies were counted using a stereomicroscope.

### 2.2. Culture Conditions of MDBK Cells

The MDBK cells were grown in Dulbecco’s Modified Eagle Medium (DMEM) supplemented with 1 g/L D-glucose, L-glutamine, and 110 mg/L sodium pyruvate. Antibiotic-free DMEM was enriched with heat-inactivated horse serum (Gibco, USA) to a final concentration of 5%. Cells were grown in a T150 flask (Falcon, Chicago, IL, USA) and maintained at 37 °C and 5% CO_2_. After inspecting for cell confluence and uniformity under microscope, cultures were washed with 1x phosphate-buffered saline (PBS, pH 7.4), then treated with 3 mL of 1x trypsin (Trypsin-EDTA, Gibco-USA) at RT for 2 min to allow the cells to detach from the flask surface.

Absence of mycoplasma contamination was confirmed by PCR. DNA extracted from noninfected MDBK cells was used as a template for the PCR mixture. The negative control consisted of DMEM without DNA template with the positive controls having DNA extracted from pure *Mycoplasma* spp. cultures. Thermal cycling was performed for 2 min at 95 °C, followed by a series of 30 cycles of 30 s at 95 °C, 20 s at 60 °C, 25 s at 72 °C, and 72 °C for 1 min. PCR was performed using the Eppendorf Mastercycler nexus GX2 thermocycler (Sigma-Aldrich, Steinheim, Germany). PCR products were visually inspected after undergoing electrophoresis at 100 V for 50 min in a 1% (*w*/*v*) agarose gel.

### 2.3. Cell Infection and Gentamicin Protection Assay

The cell infection and gentamicin protection assays were performed as previously described [34,36] but with slight modifications. Seeding was performed 24 h before infection with 1 × 10^4^ of freshly trypsinized MDBK cells added to 0.5 mL of DMEM in each well of a 24-well plate (Falcon, USA). MDBK cells were then infected for 3 h with bovine mycoplasma cultures. Since culture titers varied (ranging from 10^7^ to 10^11^ CFU/mL), different dilutions were made to determine the volume of each strain inoculum to be added to MDBK cells to arrive at a MOI of 10. The plates were shaken vigorously and washed twice with 1x PBS at pH 7.4. Extracellular mycoplasmas were killed using the gentamicin treatment, which involved an additional 3 h incubation in fresh DMEM supplemented with 400 μg/mL gentamicin (Gibco, USA). Gentamicin was removed by PBS washing and fresh DMEM added to all wells. Sample processing was performed at 6 and 54 h post-infection, which entailed PBS washing, scraping, lysing, and then centrifugation at 14,000 rpm for 10 min. Cell lysates were resuspended in 300 µL PBS, vortexed for 10 sec, and used for culture and DNA quantification by qPCR. Control cultures of noninfected MDBK cells were seeded and grown in the same conditions as described. Cell infection with all 14 *mycoplasma* strains was tested in triplicate and in three independent trials.

### 2.4. Assessment of Mycoplasmas Survival in Cell Lysates by Culture

Samples (50 µL) of the cell lysates obtained at 6 and 54 h post-infection were inoculated into PPLO broth (as described) supplemented with penicillin G and thallium acetate and incubated at 37 °C in 5% CO_2_. The positive control was individual mycoplasmas revived from glycerol stocks and the negative control was mycoplasma-free medium. Growth of mycoplasmas in cell lysates was assessed visually over a 7 d period with positive cultures confirmed by plating onto PPLO agar plates and examining for typical mycoplasma fried egg-shaped colonies using a stereomicroscope.

### 2.5. DNA Extraction, Target Genes, and Real-Time PCR (qPCR) Conditions

DNA extraction was performed on the remaining 250 µL of each cell lysate using the DNeasy Blood and Tissue DNA isolation kit (Qiagen, Hilden, Germany), as per the manufacturer’s instructions. The same extraction procedure was performed on cultures of the *M. bovis* type strain (ATCC 25523), *M. bovirhinis* field strain (MP 212-A NTC 3), *M. bovigenitalium* type strain (ATCC 19852), and the noninfected MDBK cells. DNA was quantified by the NanoDrop One spectrophotometer (Thermo Scientific, Waltham, MA, USA).

For the qPCR, three genes were selected based on specificity and conservation. For *M. bovis*, a pair of primers targeted a 92-bp amplicon of the *uvrC* housekeeping gene, as per Andres-Lasheras et al. [42]. The universal eubacterial *16S rRNA* gene, for which specificity to the domain *Bacteria* has already been confirmed, was used for the detection of *M. bovirhinis* and *M. bovigenitalium* [43]. And for quantification of the MDBK cells, the bovine *18S rRNA* gene was targeted as it is used as a reference gene for cattle [44]. Specific details about these genes and their primers are found in Table S1.

qPCR was performed using the Agilent AriaMx instrument and Luna Universal PCR Master Mix (BioLabs, Hitchin, New England). The qPCR cycling conditions were as follows: 95 °C for 1 min, then 40 cycles at 95 °C for 15 s, and at 60 °C for 30 s. Standard curves were generated from triplicate qPCR reactions using serial-diluted DNA isolated from pure cultures. Mean Cq values and the log of the DNA amounts were used to generate the standard curves using NEBioCalculator^®^ version 1.15.3 (https://nebiocalculator.neb.com/#!/qPCRGen, accessed on 30 January 2023). A standard curve was considered validated if the amplification efficiency of the target gene was within the recommended range (90–110%) and the correlation coefficient (R^2^) value approximated 1. qPCR reactions were performed in triplicate and the mean Cq used to estimate the quantity of DNA determined from the standard curves (Figure S1). Primer specificity was evaluated by the generation of a melt curve with single peak indicating a single PCR product. Amplification plots and melt curves were created in Agilent Aria software (version 1.71).

### 2.6. Calculation of the Ratio of Mycoplasma spp. to MDBK Cells

The Myc/Cell ratio relates to the number of *Mycoplasma* spp. genomes associated with each MDBK cell, providing a proxy for the infectivity potential of each strain. A multiple-step process was used to calculate the Myc/Cell ratio.

First, the number of mycoplasma and MDBK genomes in each sample and at each post-infection time (t) (6 and 54 h) was calculated as follows:


(1) $$\mathrm{Number}\;\mathrm{of}\;\mathrm{genomes}\;(sample,t)=\frac{Weight\;of\;\Sigma \;genome\;copies\;(sample,\;t)}{Weight\;of\;1\;genome\;copy\;}$$

Weight of Σ genome copies represents the mass (ng) of DNA estimated by qPCR testing at each post-infection time (t) (6 and 54 h) for one organism (mycoplasma or bovine). This quantity was determined referring to the standard curve for each target gene by converting the average Cq values per sample to weight (ng). The respective weights were then divided by the number of copies of each target gene in each *Mycoplasma* spp. and MDBK genome. *M. bovis* has one copy of the *uvrC* gene whereas *M. bovigenitalium* and *M. bovirhinis* genomes have two and three copies of the eubacterial *16S rRNA* gene, respectively. The MDBK genome has two copies of the *18S rRNA* gene. Because each genome is of different size (Mb) and weight, the next step converted the weight of the genomes to the number of genomes. Using the NCBI database (https://www.ncbi.nlm.nih.gov/), the size (Mb) of each genome was estimated and then divided by 978 Mb, which is equivalent to 1 pg [45]: *M. bovis* PG45 (Ref Seq: NC_014760.1), 1.003404 Mb; *M. bovirhinis* NCTC10118 (Ref Seq: NZ_LR214972.1), 0.901941 Mb; *M. bovigenitalium* NCTC10122 (Ref Seq: NZ_LR214970.1), 0.841512 Mb; and *Bos taurus* (cattle) (Ref seq: NC_037328.1-NC_006853.1), 2711.21 Mb. This resulted in the number of genomes of *Mycoplasma* spp. and MDBK per sample.

The Myc/Cell ratio is the ratio of *Mycoplasma* spp. genomes to MDBK genomes, which is equivalent to the number of mycoplasma per MDBK cell:

(2) $$\frac{Myc}{Cell}ratio\;(mycoplasma\;strain,\;sample,\;t)=\frac{Number\;of\;mycoplasma\;strain\;genomes\;(sample,\;t)}{Number\;of\;bovine\;genomes\;(sample,\;t)}$$

### 2.7. Confocal and Scanning Electron Microscopy Analysis

A single *M. bovis* strain (mNeonGreen) was used for the confocal (CM) and scanning electron microscopy (SEM) studies. These studies were conducted as an orthogonal means of demonstrating the adhesion and subsequent replication of *M. bovis* cells within the MDBK cells.

The CM cell infections were conducted as described above, but the cells cultured on 12 mm-diameter glass coverslips (Fisher Scientific, Schwerte, Germany) with noninfected cells were used as the negative control. Following infection, the MDBK cells were stained with 1.5× CellMask™ Deep Red Plasma Membrane Stain (Invitrogen™, Waltham, MA, USA), fixed with 4% Paraformaldehyde Aqueous Solution (Electron Microscopy Sciences, Hatfield, PA, USA), and stained with 2 µg/mL Hoechst 33258 Pentahydrate dye solution (Invitrogen™, USA), as previously described [23]. Coverslips were mounted with Prolong Diamond Antifade Mountant (Invitrogen™, USA) and observed using a confocal laser scanning microscope (TCS SP5, Leica, Wetzlar, Germany). Images were acquired with a Plan-Apochromat 63.0X 1.4 oil objective at a resolution of 512 × 512 pixels, and analyzed using the ImageJ software 1.53t (https://imagej.net/ij/, National Institutes of Health, USA).

The MDBK cells used in the SEM study were cultured on glass coverslips and infected with the *M. bovis* mNeonGreen strain at an MOI of 10 for 24 h. Infected cells were washed with PBS, treated with gentamicin, and incubated for an additional 24 h. After PBS washes, cells were fixed with 2% glutaraldehyde and 0.1 M sodium cacodylate (NaCac) buffer, pH 7.4 for 1 h at RT, and washed twice with 0.1 M NaCac, pH 7.4. Samples were then fixed in 1% osmium tetroxide (OsO4) and 0.1 M NaCac for 15 min. After NaCac washing and dH_2_O rinsing, samples were dehydrated in an ethanol gradient (30%, 50%, 70%, 80%, 90%, 95%, and 100%). In each ethanol bath, samples were dehydrated once for 5 min, with an exception for the last dehydration step, which was repeated three times. Critical point drying of samples was achieved by submersion in baths of hexamethyldisilazane (HMDS) for 5 min each (1:2 HMDS:Ethanol, 2:1 HMDS:Ethanol, and 100% HMDS three times). Finally, the coverslips were mounted on aluminum stubs and coated with 10 nm gold with a sputter coater (Quorum Q150T ES, Quorum, East Grinstead, UK). Samples were examined using a Hitachi SU8010-based scanning electron microscope (HITACHI, Tokyo, Japan) using an accelerating voltage of 3 kV.

### 2.8. Impact of the Variation of Infection Conditions

The consistency of the gentamicin protection assay was assessed by comparing mycoplasma cell infection results obtained after variation of some infection parameters (MOI and infection time) and analysis with two different techniques (qPCR and CM). This was performed using the *M. bovis* mNeonGreen strain. The MOI was changed from 10 to 50 and infection times before the gentamicin treatment increased from 3 to 24 h. Cell infection, qPCR, and CM were all conducted as described above.

## 3. Results

### 3.1. Biological and Molecular Evidence of Replication of Bovine Mycoplasma spp. Within MDBK Cells

Mycoplasmas were recovered from all MDBK cell lysates. Growth of mycoplasmas appeared earlier in lysates obtained from cells that had been incubated for 54 h vs. 6 h (Table S2). However, all 14 strains had obvious growth by day 5 regardless of the initial incubation time.

**Figure 1:**
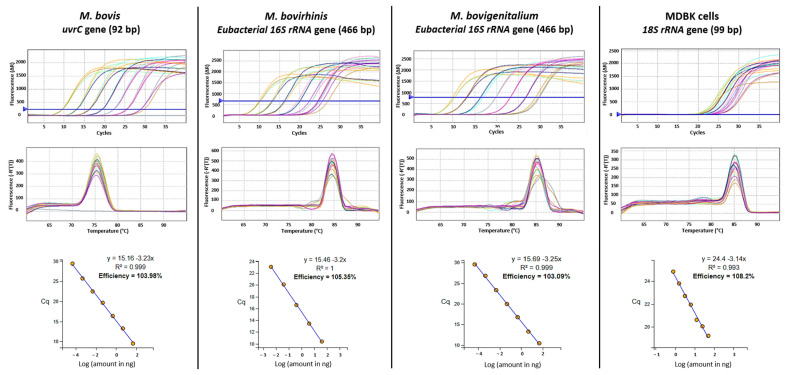
Amplification plots (**upper row**), melt curves, and qPCR standard curves (**bottom row**) generated from *Mycoplasma bovis*, *Mycoplasma bovirhinis*, *Mycoplasma bovigenitalium*, and the Madin–Darby Bovine Kidney (MDBK) cell line. The different colors correspond to the qPCR reactions using serial-diluted DNA.

Survival and intracellular growth of the mycoplasma strains were confirmed by qPCR of the MDBK cell lysates. The mycoplasma target genes were successfully amplified in DNA templates extracted from infected cell lysates collected post-gentamicin treatment (Figure S2 and S4A). Four standard curves, corresponding to the three bovine mycoplasma species as well as MDBK cells, were generated and validated. The amplification efficiency of the targeted genes ranged from 103.09% to 108.2%. Correlation coefficient (R^2^) values were between 0.993 and 0.999, and a single melting peak was observed in all qPCR reactions (Figure 1).

**Figure 2:**
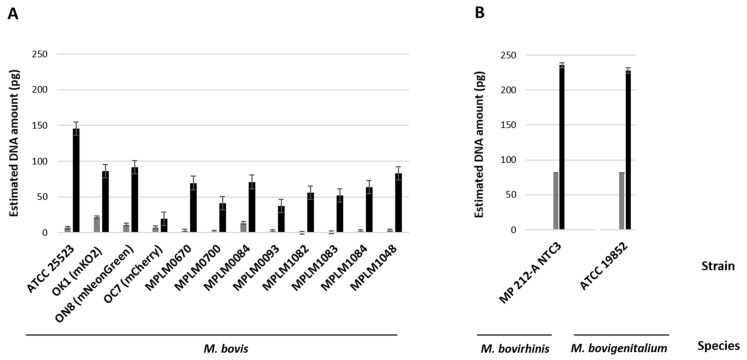
Molecular confirmation (qPCR) of bovine mycoplasma cells within MDBK cells post-gentamicin treatment. Grey and black bars represent the amount of bovine mycoplasma DNA (pg) extracted from cell lysates collected 6 h and 54 h post-infection, respectively. (**A**) Estimation of *Mycoplasma bovis* DNA using qPCR targeting the *uvrC* gene. (**B**) Estimation of DNA from *Mycoplasma bovirhinis* and *Mycoplasma bovigenitalium* using qPCR targeting the eubacterial *16S rRNA* gene. Standard deviations are indicated as vertical bars.

**Figure 3:**
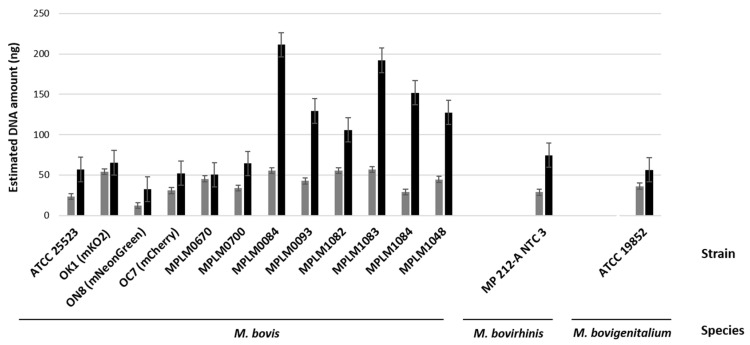
Quantitation of MDBK DNA (ng) extracted from cell lysates collected after 6 h (grey bar) and 54 h (black bar) post-infection with mycoplasma strains, and measured by qPCR (bovine *18S rRNA* gene). Standard deviations are indicated as vertical bars.

Nucleic acid amounts increased between 6 and 54 h, confirming the multiplication of mycoplasmas within the MDBK cells. *M. bovis* DNA amounts varied by strain from 0.24 to 21.91 pg at 6 h and 19.97 to 145.9 pg at 54 h (Figure 2A). The *M. bovirhinis*-type strain produced 81.75 and 235.4 pg of DNA at 6 and 54 h, respectively. *M. bovigenitalium* DNA was estimated at 81.43 pg and 227.7 pg at 6 and 54 h, respectively (Figure 2B). The bovine *18S rRNA* gene was also amplified in all samples (Figure S3 and S4B) and the deduced DNA amounts were shown to increase with incubation time (Figure 3).

### 3.2. Myc/Cell Ratios

The Myc/Cell ratios calculated at 6 and 54 h post-incubation confirmed that mycoplasmas were able to replicate within the MDBK cells. However, some differences were noted by species and origin (laboratory or field). The *M. bovirhinis* and *M. bovigenitalium* Myc/Cell ratios exceeded the *M. bovis* ratios whereas the *M. bovis* laboratory strains’ ratios were greater than those of the *M. bovis* field strains (Table 2).

### 3.3. Microscopy Studies

**Figure 4:**
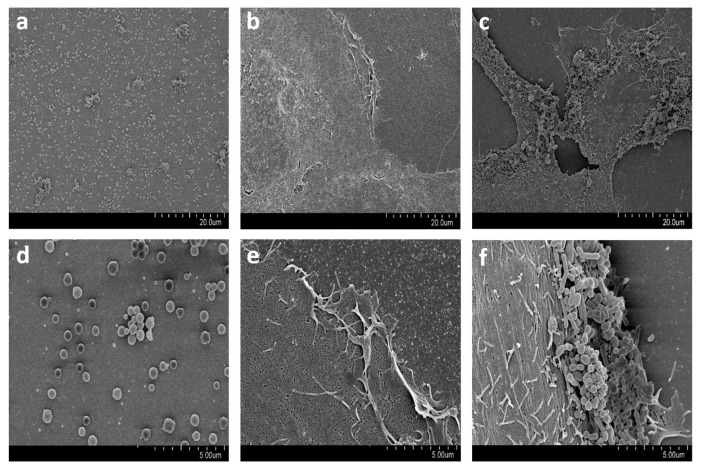
Scanning electron microscopy (SEM) photomicrographs of Madin–Darby Bovine Kidney (MDBK) cells infected with *Mycoplasma bovis* mNeonGreen strain. (**a**,**d**) SEM showing the spherical shape of *M. bovis*, cultured in the absence of MDBK cells, detected individually and in aggregates. (**b**,**e**) noninfected MDBK cells with typical surface projections. (**c**,**f**) SEM with clusters of *M. bovis* adhering to MDBK cell surface.

SEM imaging showed the adherence of *M. bovis* to the MBDK cell surface (Figure 4). CM imaging found the *M. bovis* mNeonGreen strain both extra- and intracellularly (Figure S5).

### 3.4. Variation of Myc/Cell Ratios According to Infection Conditions

Using an infection time of 3 h and an MOI of 10, the Myc/Cell ratio increased from 5.0 at 6 h to 15.2 at 54 h. Whereas an MOI of 50 resulted in Myc/Cell ratios of 93.8 to 196.9 at 6 h and 54 h, respectively. Furthermore, increasing the infection time from 3 h to 24 h pre-gentamicin treatment resulted in Myc/Cell ratios of 126.1 for MOI 10 and 450.4 for MOI 50 (Figure S6). There was a variation in fluorescence intensity according to infection conditions as shown in the confocal micrographs of MDBK cells infected with the *M. bovis* mNeonGreen strain (Figure S7), The number of green fluorescent particles, corresponding to the *M. bovis* mNeonGreen strain associated (adhesion/invasion) to MDBK cells, increased with higher MOI and longer incubation time.

## 4. Discussion

Although previous reports have documented *Mycoplasma* spp. invading eukaryotic cells, our study was unique from a number of perspectives. Previous studies have tended to focus on *M. bovis*, whereas we also included *M. bovirhinis* and *M. bovigenitalium*, which are not generally considered as significant bovine pathogens. Prior testing of *M. bovirhinis* and *M. bovigenitalium* strains in similar cell infection experiments was conducted decades ago [40,41,46]. Additionally, the MDBK cell line has been used in cell infection assays of different mycoplasmas [36,37,38], but our study was the first to infect these host cells with *M. bovirhinis* and *M. bovigenitalium*. We also tested 12 different *M. bovis* strains, which was more than double the number used in most previous studies [31,32,33,34,35,36], with the exception of one study that used 18 *M. bovis* strains [38]. Although the *M. bovis*-type strain (PG45) has been used to infect bovine mammary gland [35] and synovial cells [39], our study was one of only a few in which the PG45 strain was used to infect MDBK cells. Lastly, it is noteworthy that this study was the first to use qPCR with a gentamicin protection assay. Our qPCR technique proved reliable and efficient and served to demonstrate that the number of cell-associated mycoplasmas was dependent on incubation time and MOI.

The gentamicin protection assay has been shown in previous experiments to be a reliable method for investigating cell adhesion and invasion. Josi et al. [36] combined this assay with confocal fluorescent microscopy to show that *M. bovis* recovered from a dairy cow with pneumonia and mastitis could cause apoptosis of MDBK cells. In our case, the gentamicin protection assay served to confirm adhesion and invasion through the detection of mycoplasmas by culture and for the first time by qPCR technique. Unlike other studies [32,34,36,39], we did not use culture to quantify mycoplasmas. Rather, culture was only used to determine mycoplasma growth.

We hypothesized that the more ‘virulent’ *M. bovis* strain obtained from lesions of animals that had died of mycoplasmosis would adhere to, invade, and replicate in the host MDBK cells at greater levels than the ‘nonvirulent’ strains, which included the *M. bovirhinis*, *M. bovigenitalium*, and *M. bovis* strains from asymptomatic animals. However, the ability of the *M. bovis* strains isolated from dead and asymptomatic animals to invade and replicate within MDBK cells was similar. Also, both *M. bovirhinis* and *M. bovigenitalium* species, often reported to be apathogenic, not only adhered to bovine host cells but were also able to multiply within them. Since all *Mycoplasma* strains were equally able to adhere, invade, and multiply within the MBDK host cells, other factors must play a more significant role in whether the opportunistic pathogens cause disease or behave as commensal organisms. For example, Premachandre et al. found that genes mainly coding for ABC transporters, proteins involved in carbohydrate, nucleotide and protein metabolism, as well as membrane proteins with adhesin properties were essential for the survival of *M. bovis* in infected animals [47]. In determining the virulence of these *Mycoplasma* spp., different factors should be considered including the host susceptibility, effectiveness of the immune system, overall health status, and genetics [48,49].

While all mycoplasmas replicated within the MDBK cells, there were differences in the Myc/Cell ratios by species, MOI, duration of infection, and origin (laboratory or field). For instance, *M. bovis* laboratory strains had a mean Myc/Cell ratio that was marginally higher than the *M. bovis* field strains at both post-incubation times. A limitation of our study was that the in vitro conditions (media composition, temperature, pH) may have favoured the growth of the laboratory strains that had adapted to laboratory conditions [50]. Thomas et al. reported that virulent *M. bovis* strains adhered more than the less virulent or apathogenic strains [38]. However, *M. bovirhinis* and *M. bovigenitalium*, which are supposedly less virulent species, were equally as efficient as *M. bovis* in adhering/invading MDBK cells. Perhaps the pathophysiological characteristics of *M. bovis* strains have changed since their first isolation due to laboratory conditions or multiple passages. The field strains used here were isolated at different times over a 10-year period. Acknowledging that the virulence potential of *M. bovis* is not emergent and has been known for decades, there could be some divergent characteristics between strains in this study and between those currently circulating in livestock. Considering that bacteria species can easily adapt to changes in their environment (presence of antibiotics for example) and adjust their virulence strategies accordingly [51], surveillance of virulence in *M. bovis* strains will always be a dynamic effort. While an impact of isolation year of the field strains was not observed within this study, tracking their virulence across decades would be an interesting future endeavour.

Both confocal and electron microscopy confirmed the association of the mNeonGreen strain to MDBK cells by means of adhesion, a finding that has been previously reported [34,36]. Adhesion is considered the first step in cellular infection [52], which is facilitated by surface proteins, referred to as adhesins [52,53]. The membrane surface-exposed protein P26 has been shown to be a major adhesin of *M. bovis* [54,55]. Variable surface lipoproteins (Vsps) also have a role in *M. bovis* cell adhesion [54]. Moreover, in vitro assays show that *M. bovis* adhesion is influenced by several parameters such as cell line, temperature, MOI, and time [25,54]. In our study, all cell infection assays were performed using a single cell line (MDBK) and at one temperature (37 °C). Varying MOI showed that the number of bacteria associated with MDBK cells is dependent on the number of bacteria used for infection. Incubation time was also an important determinant with the number of cell-associated bacteria increasing with time (6 h and 54 h). While our first infection time-point was 6 h, adhesion and invasion of *M. bovis* PG45-type strain into bovine synovial cells has been reported to occur within 15 min post-infection [39].

The finding that all three bovine mycoplasmas were able to invade the MDBK cells is consistent with numerous other animal and human mycoplasmas such as *M. agalactiae*, *M. suis*, *M. hyopneumoniae*, *M. synoviae*, *M. genitalium*, *M. fermentans*, *M. pneumoniae*, and *M. hominis* [56,57,58,59,60,61,62,63,64]. Different invasion patterns were found as these *Mycoplasma* spp. have been detected intracellularly in several locations such as within the cytoplasm, within and around the nucleus, on the cytosolic side of cell membranes, and within vacuole-like structures [34,56,57,58,59,60,61,62,63,64]. In our study, *M. bovis* was found adhering to MDBK extracellularly and also visualized in the cytoplasm. Invagination and disruption of the host cell membrane after infection as well as localization of *M. bovis* within vesicle-like structures have been previously reported [34,39]. These events and structures have been associated with endocytosis [65,66], with Bürki et al. speculating that endocytosis is the pathway by which bovine mycoplasmas enter epithelial cells [34].

The use of CM and SEM confirmed the existence of mycoplasmas located intracellularly and extracellularly. Mycoplasma cells found adhered to the outer MDBK cell membrane appeared to have survived the gentamicin treatment despite its proven effectiveness to kill extracellular mycoplasmas [36]. Josi et al. reported similar findings using a gentamicin protection assay, MDBK cells, post-infection times of 6 and 54 h, and confocal microscopy [36]. The reproducibility of this finding confirms that mycoplasmas can be found adhering to the host cells even after gentamicin treatment. It is hypothesized that cell lysis and the subsequent release of intracellular mycoplasmas may account for the extracellular *M. bovis* cells [36]. Alternatively, Hedge et al. working with *M. agalactiae*, hypothesized that extracellular mycoplasmas result from an evasion event following invasion of host cells [56]. This latter supposition can be supported by previous studies wherein invasion and evasion events were involved in the dissemination of mycoplasmas within the host [67,68,69,70]. We found that most extracellular *M. bovis* were in contact with the MDBK cells and rarely alone in cell-free areas, which may be related in part to these organisms being obligate parasites and hence fusion with the host membrane may be a nutritional requirement [71].

## 5. Conclusions

Our findings confirm that *M. bovis*, *M. bovirhinis*, and *M. bovigenitalium* can invade, persist, and multiply in bovine kidney cells. The ability to persist intracellularly allows the organism to evade the host’s immune system. Overall, the adhesion and invasion into MDBK cells occurred independently of whether they originated from asymptomatic or diseased animals or were bovine *Mycoplasma* spp., which are generally considered less pathogenic. While adherence, invasion and replication within host cells are critical stages in the pathogenesis of mycoplasmosis, it appears that the virulence genes required for these steps are highly conserved across mycoplasma species. Thus, in vitro experiments alone may not be suitable for assessing the infectivity and pathogenicity of mycoplasma strains since they may not replicate the complex dynamics of the host microbiome, amongst other factors in natural infections. At a minimum, the study of the host–pathogen interaction and the evaluation of mycoplasmal infection impact on cells should be based on more mycoplasma species/strains, considering more virulence attributes, and using different cell lines (such as bovine turbinate cells).

## Figures and Tables

**Table 1:** Metadata associated with the *Mycoplasma* strains used in this study to infect Madin–Darby Bovine Kidney (MDBK) cells and to assess adhesion and invasion characteristics.

			Country of Origin	Host Species	Health Status	Anatomical Isolation Site	Isolation Year
*M. bovis*	Type strain	PG45 (ATCC 25523)	USA	Cattle	Mastitis	Milk	1962
	Fluorescent strains	OK1 (mKO2)	France *	Cattle	Diseased	Lung	1975/2016 **
		ON8 (mNeonGreen)			Diseased	Lung	
		OC7 (mCherry)			Diseased	Lung	
	Field strains	MPLM0670	Canada	Cattle	Asymptomatic	Nasopharynx	2007
		MPLM0700				Nasopharynx	2007
		MPLM0084			Dead	Lung	2017
		MPLM0093				Lung	2017
		MPLM1082		Bison	Asymptomatic	Nasopharynx	2014
		MPLM1083				Nasopharynx	2014
		MPLM1084			Dead	Lung	2015
		MPLM1048				Joint	2011
*M. bovirhinis*	Field strain	MP 212-A NTC 3	Canada	Cattle	Dead	Lung	2014
*M. bovigenitalium*	Type strain	NCTC 10122 (ATCC 19852)		Cattle	Unknown	Bovine genital tract	1955

* The three modified *M. bovis* Oger2 strains were provided by Bennefois et al. from CIRAD, Montpelier-France [23]; ** *M. bovis* Oger2 strain was isolated in 1975. From this strain, *M. bovis* OK1, ON8, and OC7 fluorescent strains were engineered in 2016 [23].

**Table 2:** Myc/Cell ratios by *Mycoplasma* strains (n = 14) with mean ratios calculated by species and groups.

Species	Group	*Mycoplasma* Strain	Myc/Cell Ratio (6 h)	Myc/Cell Ratio (54 h)
*M. bovis*	Laboratory strains	PG45 (ATCC 25523)	1.7	13.9
		OK1 (mKO2)	2.2	7.1
		ON8 (mNeonGreen)	5.0	15.2
		OC7 (mCherry	1.3	2.1
	**Mean value of Myc/Cell ratios**		**2.5**	**9.6**
	Field strains	MPLM0670	0.3	7.4
		MPLM0700	0.3	3.5
		MPLM0084	1.3	1.8
		MPLM0093	0.3	1.6
		MPLM1082	0.02	2.8
		MPLM1083	0.04	1.5
		MPLM1084	0.4	2.3
		MPLM1048	0.4	3.5
	**Mean value of Myc/Cell ratios**		**0.4**	**3.0**
**Mean value of Myc/Cell ratios**			**1.1**	**5.2**
*M. bovirhinis*		MP 212-A NTC 3		
**Myc/Cell ratio**			**5.6**	**6.2**
*M. bovigenitalium*		NCTC 10122 (ATCC 19852)		
**Myc/Cell ratio**			**7.3**	**13.1**

## Data Availability

The original contributions presented in this study are included in the article/Supplementary Material. Further inquiries can be directed to the corresponding author.

## Supplementary Materials

The following supporting information can be downloaded at: https://www.mdpi.com/article/10.3390/microorganisms13030632/s1, Figure S1. Flowchart explaining the qPCR reactions performed to amplify 2 genes (mycoplasma and bovine) at 2 post-infection times (6 h and 54 h) in DNA extracted from cell lysates recovered after MDBK cell infection with 14 different mycoplasma strains. Infection was repeated in the three independent trials. At each trial, each sample was treated in triplicate at each post-infection time. The mean Cq values were used to estimate the quantity of DNA in each sample based on the corresponding standard curve. Figure S2. qPCR amplification of *uvrC* gene after MDBK cells infection with *Mycoplasma bovis* strains. Amplification plots resulted from triplicate qPCR using DNA extracted from cell lysates collected after 6 h and 54 h in three independent infection experiments. For each sample at each time point and at every trial, mean Cq values were calculated from triplicate qPCR reactions. Box and whisker plots show the variation of mean Cq values of the different samples. Amplification plots and melt curves were generated using Agilent Aria software version 1.71. Figure S3. qPCR amplification of the bovine *18S rRNA* gene after MDBK cells infection with *Mycoplasma bovis* strains. Amplification plots resulted from triplicate qPCR using DNA extracted from cell lysates collected after 6 h and 54 h in three independent infection experiments. For each sample at each time point and at every trial, mean Cq values were calculated from triplicate qPCR reactions. Box and whisker plots show the variation of mean Cq values of the different samples. Amplification plots and melt curves were generated using Agilent Aria software version 1.71. Figure S4. qPCR amplification of eubacterial *16S rRNA* gene (**A**) and bovine *18S rRNA* gene (**B**) after MDBK cells infection with *Mycoplasma bovirhinis* and *Mycoplasma bovigenitalium* strains. Amplification plots resulted from triplicate qPCR using DNA extracted from cell lysates collected after 6 h and 54 h in three independent infection experiments. For each sample at each time point and at every trial, mean Cq values were calculated from triplicate qPCR reactions for each gene. Box and whisker plots show the variation of mean Cq values of the different samples. Amplification plots and melt curves were generated using Agilent Aria software version 1.71. Figure S5. Confocal microscopy (CM) photomicrographs of Madin-Darby Bovine Kidney (MDBK) cells infected with *Mycoplasma bovis* mNeonGreen strain. Panel (**a**) expression of the GFP (green fluorescent protein) in *M. bovis* mNeonGreen strain confirmed prior to the CM study. Panel (**b**) noninfected MDBK cells. Panel (**c**) CM of *M. bovis* adhered extracellularly (white arrows) and within the cytoplasm (yellow arrows) of MDBK cells. The MDBK cellular membrane and cytoplasm are stained red and the cell nucleus blue. Figure S6. Myc/Cell ratio variation according to infection conditions. Assessment of mycoplasmas uptake based on qPCR results. From left to right, values correspond to infection time before gentamicin treatment, duration of treatment, and incubation time post-treatment. Figure S7. Confocal microscopy with red corresponding to the MDBK cell membrane and cytoplasm, blue the cell nucleus, and green indicates mNeonGreen fluorescent *Mycoplasma bovis* strain. (**a**) Uninfected MDBK cells. (**b**–**d**) MDBK cells infected with *Mycoplasma bovis* mNeonGreen fluorescent strain according to different conditions (MOI and time). Values at the bottom of each panel, from left to right, correspond to infection time before, during, and after gentamicin treatment. Table S1. Target genes and primers used in qPCR reactions to amplify *Mycoplasma bovis, Mycoplasma bovirhinis*, *Mycoplasma bovigenitalium*, and *Bos taurus* DNA. Table S2. Growth characteristics of 14 different *Mycoplasma* spp.: *Mycoplasma bovis* (n = 12), *Mycoplasma bovirhinis* (n = 1)*,* and *Mycoplasma bovigenitalium* (n = 1). Each strain was used to separately infect Madin-Darby Bovine Kidney (MDBK) cells for 6 or 54 h. Following infection, the cells were lysed and the lysates incubated and monitored for the presence of mycoplasma growth for up to 7 days. All infections were performed in triplicate.
