# Supplementary material for: Virulence Is More than Adhesion and Invasion Ability, an In Vitro Cell Infection Assay of Bovine Mycoplasma spp
Source: Microorganisms. 2025 Mar 11;13(3):632. doi: 10.3390/microorganisms13030632 (PMC11944293; doi:10.3390/microorganisms13030632)
Supplement: Supplementary file 1 [file microorganisms-13-00632-s001.zip › Figure S1.pdf]

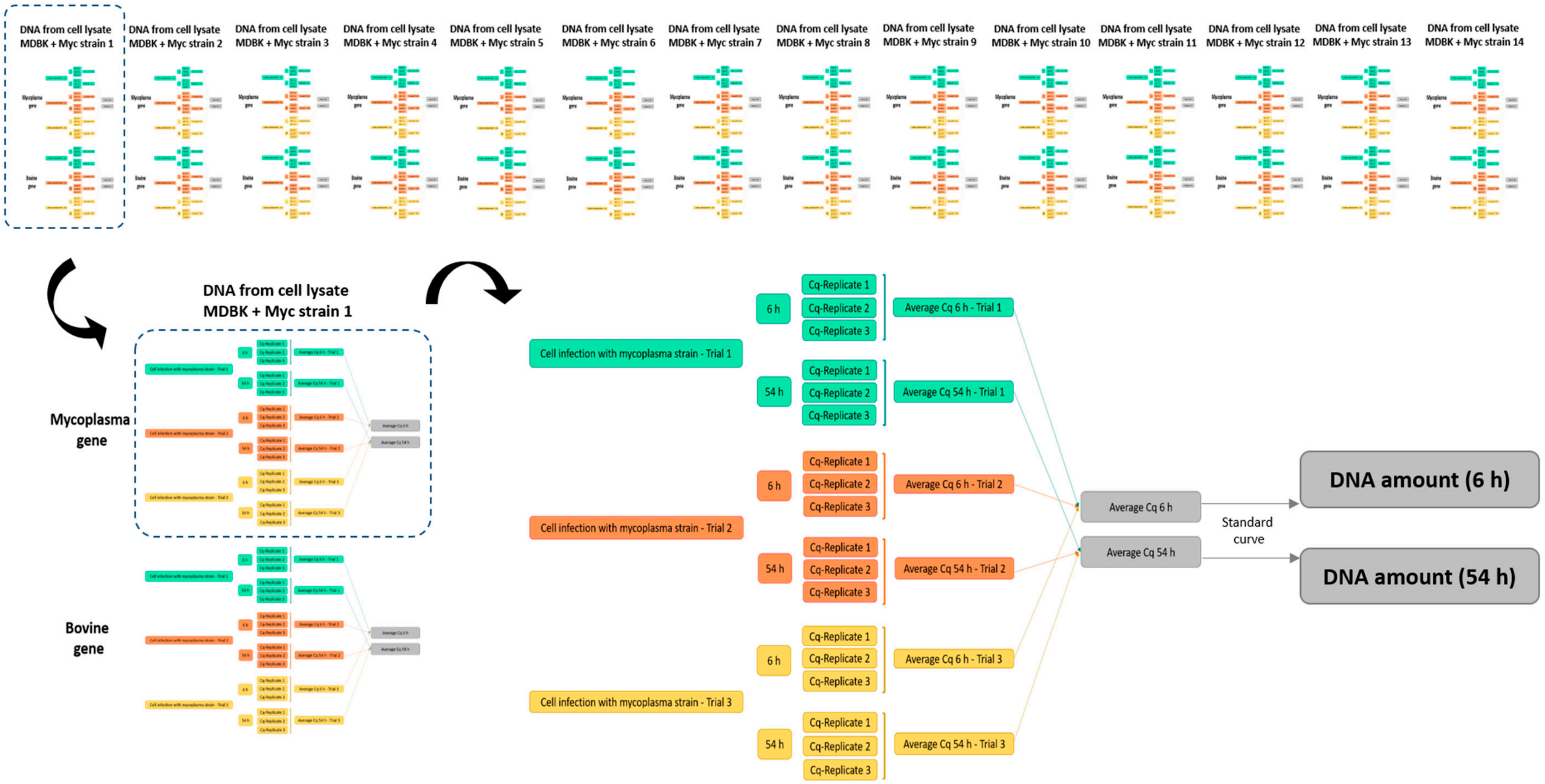

Figure S1. Flowchart explaining the qPCR reactions performed to amplify 2 genes (mycoplasma and bovine) at 2 post-infection times (6 h and 54 h) in DNA extracted from cell lysates recovered after MDBK cell infection with 14 different mycoplasma strains. Infection was repeated in the three independent trials. At each trial, each sample was treated in triplicate at each post-infection time. The mean Cq values were used to estimate the quantity of DNA in each sample based on the corresponding standard curve.
