# Supplementary material for: Virulence Is More than Adhesion and Invasion Ability, an In Vitro Cell Infection Assay of Bovine Mycoplasma spp
Source: Microorganisms. 2025 Mar 11;13(3):632. doi: 10.3390/microorganisms13030632 (PMC11944293; doi:10.3390/microorganisms13030632)
Supplement: Supplementary file 1 [file microorganisms-13-00632-s001.zip › Figure S3.pdf]

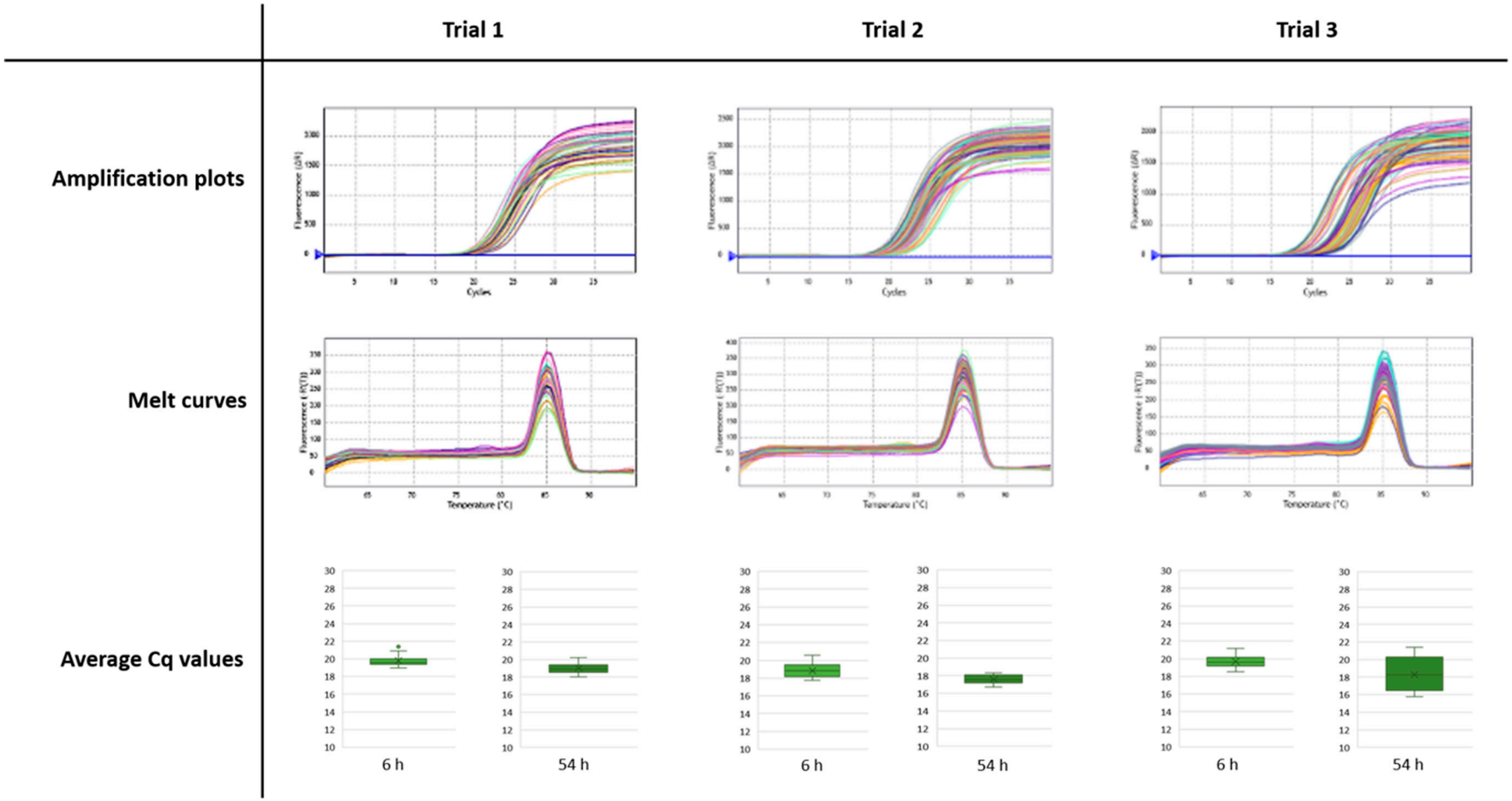


**Figure S3.** qPCR amplification of the bovine *18S rRNA* gene after MDBK cells infection with *Mycoplasma bovis* strains. Amplification plots resulted from triplicate qPCR using DNA extracted from cell lysates collected after 6 h and 54 h in three independent infection experiments. For each sample at each time point and at every trial, mean Cq values were calculated from triplicate qPCR reactions. Box and whisker plots show the variation of mean Cq values of the different samples. Amplification plots and melt curves were generated using Agilent Aria software version 1.71.
