# Supplementary material for: Virulence Is More than Adhesion and Invasion Ability, an In Vitro Cell Infection Assay of Bovine Mycoplasma spp
Source: Microorganisms. 2025 Mar 11;13(3):632. doi: 10.3390/microorganisms13030632 (PMC11944293; doi:10.3390/microorganisms13030632)
Supplement: Supplementary file 1 [file microorganisms-13-00632-s001.zip › Figure S5.pdf]

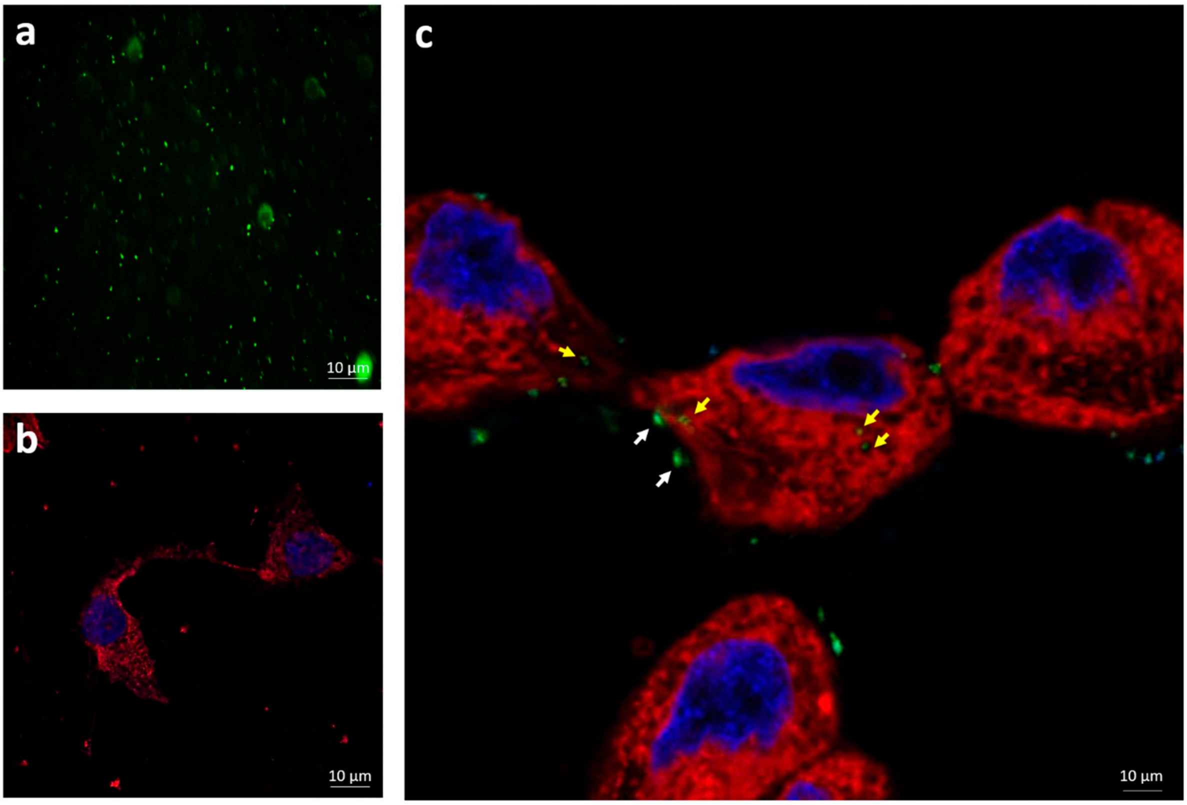

Figure S5. Confocal microscopy (CM) photomicrographs of Madin-Darby Bovine Kidney (MDBK) cells infected with *Mycoplasma bovis* mNeonGreen strain. **Panel a -** expression of the GFP (green fluorescent protein) in *M. bovis* mNeonGreen strain confirmed prior to the CM study. **Panel b -** noninfected MDBK cells. **Panel c -** CM of *M. bovis* adhered extracellularly (white arrows) and within the cytoplasm (yellow arrows) of MDBK cells. The MDBK cellular membrane and cytoplasm are stained red and the cell nucleus blue.
