# Supplementary material for: Virulence Is More than Adhesion and Invasion Ability, an In Vitro Cell Infection Assay of Bovine Mycoplasma spp
Source: Microorganisms. 2025 Mar 11;13(3):632. doi: 10.3390/microorganisms13030632 (PMC11944293; doi:10.3390/microorganisms13030632)
Supplement: Supplementary file 1 [file microorganisms-13-00632-s001.zip › Figure S6.pdf]

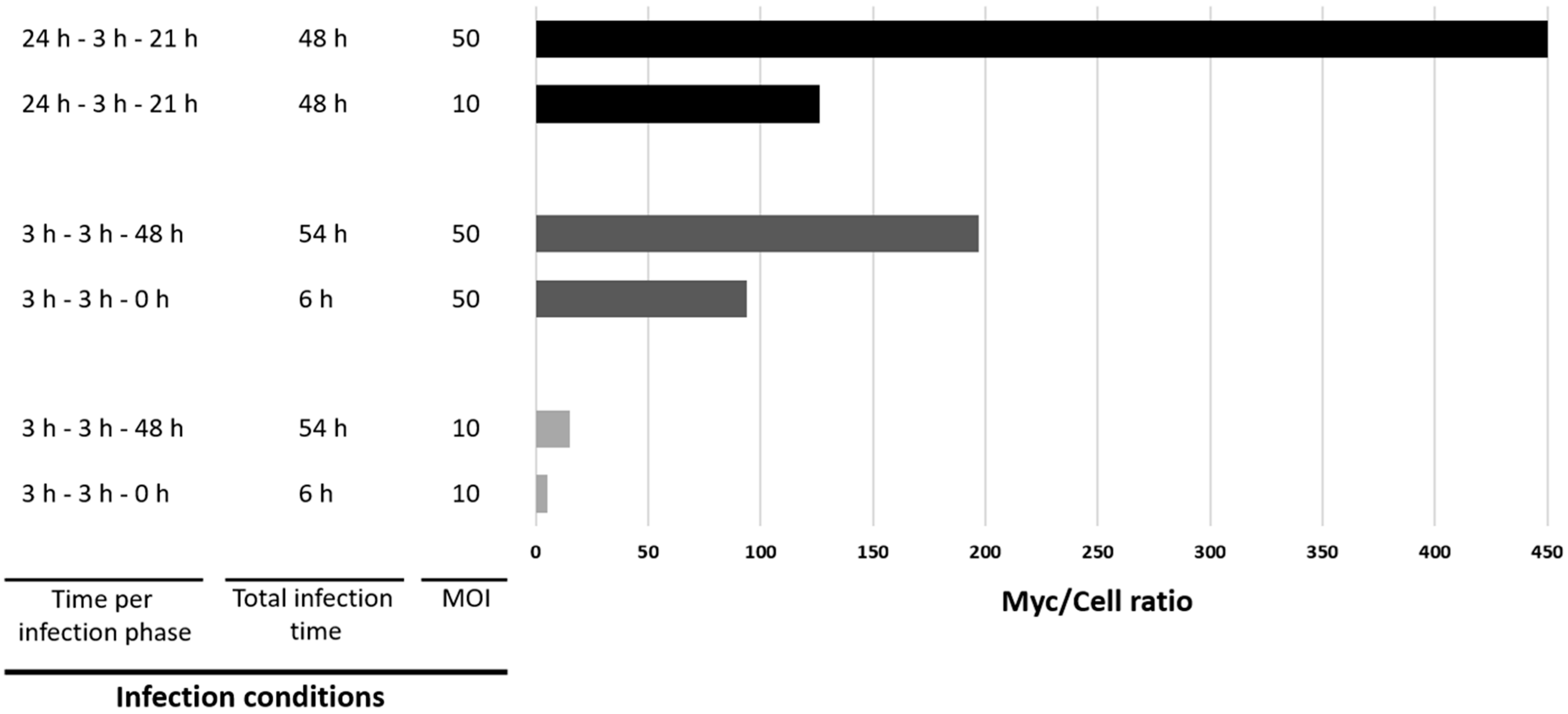

Figure S6. Myc/Cell ratio variation according to infection conditions. Assessment of mycoplasmas uptake based on qPCR results. From left to right, values correspond to infection time before gentamicin treatment, duration of treatment, and incubation time post-treatment.
