# Supplementary material for: Virulence Is More than Adhesion and Invasion Ability, an In Vitro Cell Infection Assay of Bovine Mycoplasma spp
Source: Microorganisms. 2025 Mar 11;13(3):632. doi: 10.3390/microorganisms13030632 (PMC11944293; doi:10.3390/microorganisms13030632)
Supplement: Supplementary file 1 [file microorganisms-13-00632-s001.zip › Figure S7.pdf]

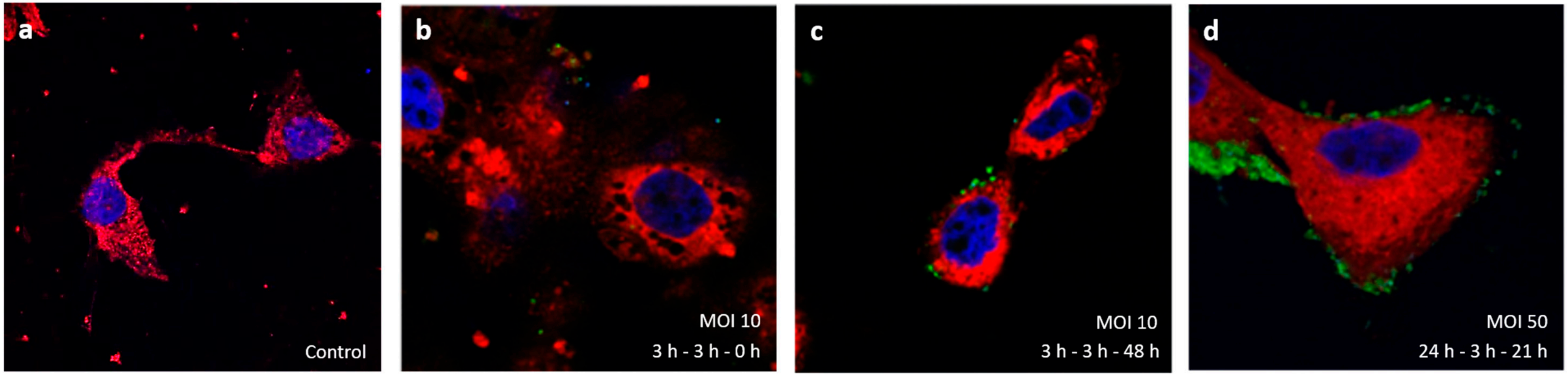

Figure S7. Confocal microscopy with red corresponding to the MDBK cell membrane and cytoplasm, blue the cell nucleus, and green indicates mNeonGreen fluorescent *Mycoplasma bovis* strain. (**a**) Uninfected MDBK cells. (**b-d**) MDBK cells infected with *Mycoplasma bovis* mNeonGreen fluorescent strain according to different conditions (MOI and time). Values at the bottom of each panel, from left to right, correspond to infection time before, during, and after gentamicin treatment.
