# Supplementary material for: Virulence Is More than Adhesion and Invasion Ability, an In Vitro Cell Infection Assay of Bovine Mycoplasma spp
Source: Microorganisms. 2025 Mar 11;13(3):632. doi: 10.3390/microorganisms13030632 (PMC11944293; doi:10.3390/microorganisms13030632)
Supplement: Supplementary file 1 [file microorganisms-13-00632-s001.zip › Table S1.pdf]

Table S1. Target genes and primers used in qPCR reactions to amplify *Mycoplasma bovis, Mycoplasma bovirhinis, Mycoplasma bovigenitalium,* and *Bos taurus* DNA.

**Target gene**	**Species**	**Primer sequence (5′-3′)**	**Amplicon size (bp)**	**Reference**
uvrC	*M. bovis*	Forward: CCTGTCGGAGTTGCAATTGT<br>Reverse: GCACTGCGCTCATTTAAAGC	92	**[43]**
16S rRNA	*M. bovirhinis*<br>*M. bovigenitalium*	Forward: TCCTACGGGAGGCAGCAGT<br>Reverse: GGACTACCAGGGTATCTAATCCTGTT	466	**[44]**
18S rRNA	*Bos taurus*	Forward: TTCGATGGTAGTCGCTGTGC<br>Reverse: TTGGATGTGGTAGCCGTTTCT	99	**[45]**
