# Supplementary material for: Virulence Is More than Adhesion and Invasion Ability, an In Vitro Cell Infection Assay of Bovine Mycoplasma spp
Source: Microorganisms. 2025 Mar 11;13(3):632. doi: 10.3390/microorganisms13030632 (PMC11944293; doi:10.3390/microorganisms13030632)
Supplement: Supplementary file 1 [file microorganisms-13-00632-s001.zip › Table S2.pdf]

Table S2. Growth characteristics of 14 different *Mycoplasma* spp.: *Mycoplasma bovis* (n = 12), *Mycoplasma bovirhinis* (n = 1), and *Mycoplasma bovigenitalium* (n = 1). Each strain was used to separately infect Madin-Darby Bovine Kidney (MDBK) cells for 6 or 54 h. Following infection, the cells were lysed and the lysates incubated and monitored for the presence of mycoplasma growth for up to 7 days. All infections were performed in triplicate.

			MDBK cell infection Trial 1							MDBK cell infection Trial 2							MDBK cell infection Trial 3						
**Bovine mycoplasma strains**		**Incubation time**	**Day 1**	**Day 2**	**Day 3**	**Day 4**	**Day 5**	**Day 6**	**Day 7**	**Day 1**	**Day 2**	**Day 3**	**Day 4**	**Day 5**	**Day 6**	**Day 7**	**Day 1**	**Day 2**	**Day 3**	**Day 4**	**Day 5**	**Day 6**	**Day 7**
M. bovis	ATCC 25233	6 h	-	-	±	+	+	+	+	-	±	±	+	+	+	+	-	-	±	+	+	+	+
		54 h	-	+	+	+	+	+	+	-	±	+	+	+	+	+	-	±	+	+	+	+	+
	OK1 (mKO2)	6 h	-	-	±	+	+	+	+	-	±	+	+	+	+	+	-	-	+	+	+	+	+
		54 h	±	+	+	+	+	+	+	-	+	+	+	+	+	+	-	+	+	+	+	+	+
	ON8 (mNeonGreen)	6 h	-	-	±	+	+	+	+	-	-	±	+	+	+	+	-	-	±	±	+	+	+
		54 h	-	+	+	+	+	+	+	-	±	±	+	+	+	+	-	+	+	+	+	+	+
	OC7 (mCherry)	6 h	-	±	±	+	+	+	+	-	-	-	+	+	+	+	-	-	±	±	+	+	+
		54 h	-	+	+	+	+	+	+	-	-	±	+	+	+	+	-	+	+	+	+	+	+
	MPLM0670	6 h	-	-	-	+	+	+	+	-	-	-	+	+	+	+	-	-	±	±	+	+	+
		54 h	-	±	+	+	+	+	+	-	-	±	+	+	+	+	-	+	+	+	+	+	+
	MPLM0700	6 h	-	-	-	+	+	+	+	-	-	-	+	+	+	+	-	-	±	+	+	+	+
		54 h	-	±	+	+	+	+	+	-	-	±	+	+	+	+	-	+	+	+	+	+	+
	MPLM0084	6 h	-	-	-	+	+	+	+	-	±	+	+	+	+	+	-	-	±	+	+	+	+
		54 h	-	+	+	+	+	+	+	-	+	+	+	+	+	+	-	+	+	+	+	+	+
	MPLM0093	6 h	-	-	-	+	+	+	+	-	-	±	+	+	+	+	-	-	±	+	+	+	+
		54 h	-	+	+	+	+	+	+	-	+	+	+	+	+	+	-	+	+	+	+	+	+
	MPLM1082	6 h	-	-	±	+	+	+	+	-	±	+	+	+	+	+	-	-	+	+	+	+	+
		54 h	-	+	+	+	+	+	+	-	+	+	+	+	+	+	-	±	+	+	+	+	+
	MPLM1083	6 h	-	-	±	+	+	+	+	-	±	+	+	+	+	+	-	-	+	+	+	+	+
		54 h	-	+	+	+	+	+	+	-	±	±	+	+	+	+	-	+	+	+	+	+	+
	MPLM1084	6 h	-	-	±	+	+	+	+	-	+	+	+	+	+	+	-	-	+	+	+	+	+
		54 h	-	+	+	+	+	+	+	-	±	±	±	+	+	+	-	±	+	+	+	+	+
	MPLM1048	6 h	-	-	±	+	+	+	+	-	-	±	+	+	+	+	-	-	±	+	+	+	+
		54 h	-	+	+	+	+	+	+	-	±	+	+	+	+	+	-	+	+	+	+	+	+
M. bovirhinis	MP 212-A NTC 3	6 h	-	-	±	+	+	+	+	-	-	-	±	+	+	+	-	-	±	±	+	+	+
		54 h	-	+	+	+	+	+	+	-	-	+	+	+	+	+	-	-	+	+	+	+	+
M. bovigenitalium	ATCC 19852	6 h	-	-	-	+	+	+	+	-	-	±	±	+	+	+	-	-	±	±	+	+	+
		54 h	-	±	+	+	+	+	+	-	±	+	+	+	+	+	-	±	+	+	+	+	+

(-) No growth
(±) Beginning of growth
(+) Obvious growth
